# Distinct functions of two olfactory marker protein genes derived from teleost-specific whole genome duplication

**DOI:** 10.1186/s12862-015-0530-y

**Published:** 2015-11-10

**Authors:** Hikoyu Suzuki, Masato Nikaido, Kimiko Hagino-Yamagishi, Norihiro Okada

**Affiliations:** Department of Biological Sciences, Graduate School of Bioscience and Biotechnology, Tokyo Institute of Technology, Yokohama, 226-8501 Japan; Department of Dementia and Higher Brain Function, Integrated Neuroscience Research Project, Tokyo Metropolitan Institute of Medical Science, Tokyo, 156-8506 Japan; Foundation for Advancement of International Science, Tsukuba, 305-0821 Japan; Department of Life Sciences, National Cheng Kung University, Tainan, 701 Taiwan

**Keywords:** Olfactory marker protein, Whole genome duplication, Subfunctionalization

## Abstract

**Background:**

Whole genome duplications (WGDs) have been proposed to have made a significant impact on vertebrate evolution. Two rounds of WGD (1R and 2R) occurred in the common ancestor of Gnathostomata and Cyclostomata, followed by the third-round WGD (3R) in a common ancestor of all modern teleosts. The 3R-derived paralogs are good models for understanding the evolution of genes after WGD, which have the potential to facilitate phenotypic diversification. However, the recent studies of 3R-derived paralogs tend to be based on *in silico* analyses. Here we analyzed the paralogs encoding teleost olfactory marker protein (OMP), which was shown to be specifically expressed in mature olfactory sensory neurons and is expected to be involved in olfactory transduction.

**Results:**

Our genome database search identified two *OMP*s (*OMP1* and *OMP2*) in teleosts, whereas only one was present in other vertebrates. Phylogenetic and synteny analyses suggested that *OMP1* and *2* were derived from 3R. Both *OMP*s showed distinct expression patterns in zebrafish; *OMP1* was expressed in the deep layer of the olfactory epithelium (OE), which is consistent with previous studies of mice and zebrafish, whereas *OMP2* was sporadically expressed in the superficial layer. Interestingly, *OMP2* was expressed in a very restricted region of the retina as well as in the OE. In addition, the analysis of transcriptome data of spotted gar, a non-teleost fish, revealed that single *OMP* gene was expressed in the eyes.

**Conclusion:**

We found distinct expression patterns of zebrafish *OMP1* and *2* at the tissue and cellular level. These differences in expression patterns may be explained by subfunctionalization as the model of molecular evolution. Namely, single *OMP* gene was speculated to be originally expressed in the OE and the eyes in the common ancestor of all Osteichthyes (bony fish including tetrapods). Then, two *OMP* gene paralogs derived from 3R-WGD reduced and specialized the expression patterns. This study provides a good example for analyzing a functional subdivision of the teleost OE and eyes as revealed by 3R-derived paralogs of *OMP*s.

**Electronic supplementary material:**

The online version of this article (doi:10.1186/s12862-015-0530-y) contains supplementary material, which is available to authorized users.

## Background

**Fig. 1:**
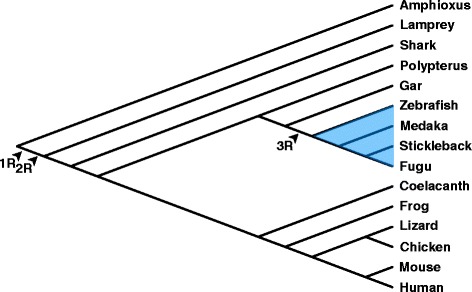
Third rounds of WGDs during vertebrate evolution. Arrowheads on the consensus phylogenetic tree of chordates indicate the timing of 1R, 2R, and 3R. The blue highlighted region indicates the teleost clade. Representative chordate species are shown

Gene duplication is one of the major driving forces of evolution [1–3]. In particular, whole genome duplication (WGD) has been thought to be an important factor in the evolution of vertebrates [1]. It has been proposed that at least two rounds of WGDs occurred during the evolution of vertebrates [1, 4, 5] (Fig. 1). Recent genome studies of amphioxus [6] and lamprey [7], support this hypothesis. The first-and second-round WGD (1R and 2R, respectively) are suggested to have occurred in the common ancestor of Gnathostomata and Cyclostomata [7, 8]. Thus, almost all modern vertebrates are believed to have undergone WGDs at least twice [1]. The third-round WGD (3R), in contrast, occurred in the common ancestor of teleosts (ray-finned fish excluding basal groups belong to polypteriforms, acipenseriforms, lepisosteids, and Amia) [8–12]. This is represented by the copy number of genes, two in teleosts, one in mammals [12, 13]. The *Hox* cluster is the most well-known example in this regard [9–11]. There are seven *Hox* clusters in teleost genomes, whereas four clusters are present in mammalian, coelacanth, and shark genomes [11]. In addition, slightly differentiated expression patterns are observed for teleost *Hox* paralogs derived from 3R [14–17]. Thus, teleost-specific duplicated genes seem to be on a path to functional differentiation, namely, 3R occurred neither too recently nor too early to differentiate the function of paralogs. 3R-derived paralogs could be good examples for evaluating the critical timing of functional differentiation.

In the case of gene duplication, it is traditionally expected that one of the duplicated genes becomes free from selective pressure and accumulates mutations in the protein coding and/or cis regulatory regions that led to the loss of its functions (pseudogenization) or gain of new functions (neofunctionalization) [2, 3]. Alternatively, subfunctionalization is observed especially as a result of WGD. In subfunctionalization, both paralogs are functional, but each paralog undergoes a complementary reduction and specialization in its expression pattern because of the mutation of its *cis* regulatory regions [3, 18, 19]. The subfunctionalized paralogs are also expected to gain new function over evolutionary time [20]. Most of the different expression patterns between teleost-specific paralogs that have been shown by previous studies [14–17, 21] could be explained by subfunctionalization. However, in these studies, the different expression patterns were usually discussed based only on *in silico* studies. In cases where analyses were carried out in vivo, many were performed only at the whole-mount tissue level. Very few comparative expression analyses have been carried out at the cellular level, which is indispensable for the investigation of subfunctionalization.

Olfactory marker protein (OMP) was first isolated from mouse olfactory bulb in the 1970s [22]. OMP is a small protein (~20 kDa) that consists of ~160 amino acids and is specifically expressed in olfactory sensory neurons (OSNs), which are distributed in the main olfactory epithelium in various vertebrates [22–24]. Although *OMP* is used as a specific marker of mature OSNs in vertebrates [25–28], its function has not yet been fully elucidated. *OMP*-knockout (KO) mice have reduced physiological activity and behavioral responses with respect to sensing odorants as compared with wild-type mice [29, 30]. In addition, axons of OSNs from *OMP*-KO mice project abnormally [31]. Recent studies have suggested that OMP is a critical factor for the functional maturation of OSNs [32] and is likely to be involved in Ca^2+^ clearance in OSNs [33]. Namely, the phenotypes observed in *OMP*-KO mice mentioned above might be caused by a decline in the clearance of Ca^2+^ in these OSNs.

*OMP* had been believed to be a highly conserved single-copy intronless gene among all vertebrates [24, 27, 34, 35]. More recently, it was shown that African clawed frog (*Xenopus laevis*) and some teleosts have two *OMP*s [36–38]. In African clawed frog, the expression patterns of the two *OMP*s are notably, albeit not completely, distinct in the lateral diverticulum and medial diverticulum, in the nasal cavity [36]. These expression patterns are suggestive of subfunctionalization. Although the expression of each of the two *OMP*s was analyzed in medaka (*Oryzias latipes*) [37] and salmon (*Oncorhynchus nerka*) [38], detailed expression patterns were not assessed. In this study, we found with a bioinformatic analysis that teleosts generally possess two *OMP*s in their genomes. Our phylogenetic analyses revealed that two OMPs are derived from 3R. Until now, expression pattern of OMP has been investigated in many vertebrates. Accordingly, we expected that detail verification of the expression patterns of OMPs could be a good example to understand the fate of 3R-derived paralogs. We here shows the detailed expression patterns of two *OMP*s at the tissue and the cellular level in zebrafish (*Danio rerio*).

## Results

### Two OMPs derived from the third-round whole genome duplication in teleosts

**Fig. 2:**
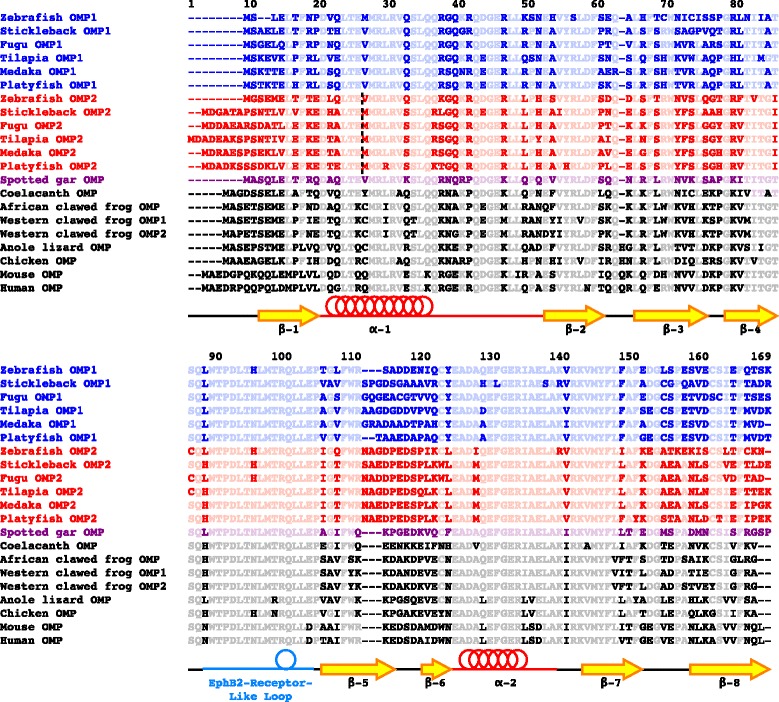
Sequence analysis of *OMP*. Alignment of amino acid sequences was constructed by ClustalW2. Colors indicate *OMP* subgroups: blue, teleost *OMP1*; red, teleost *OMP2*; purple, gar *OMP*; black, tetrapod *OMP*. Hyphens indicate gaps. Light characters indicate conserved amino acids among homologs. Vertical dashed line is the junction of *OMP2* exon 1 and exon 2. Secondary structure is based on Smith et al. [35]. OMP has two α-helical regions and eight β-pleated sheets, as shown in the structure below the sequences

To identify *OMP*s in teleost genomes, we searched genome databases of zebrafish, stickleback (*Gasterosteus aculeatus*), fugu (*Takifugu rubripes*), medaka, platyfish (*Xiphophorus maculatus*), and tilapia (*Oreochromis niloticus*) by using known *OMP* sequences as queries, and obtained two significant hits from each species (see Methods for Data mining). Although zebrafish was believed to have a single copy of *OMP* [27], we isolated two *OMP*s from the genomes of all teleost, including zebrafish. We named the already-known zebrafish *OMP* as *OMP1* and its paralog as *OMP2*. Furthermore, Ensembl gene prediction suggested that *OMP2* consists of two exons, although *OMP* is known as an intronless gene [24, 36]. We searched *OMP2* sequences from the EST database and confirmed that the predictions are consistent with the mRNA sequences in zebrafish, stickleback, and medaka. In other teleost species, we estimated the *OMP2* gene structure with GeneWise. We also searched other vertebrate genome databases and isolated *OMP* orthologs. Then, we aligned the amino acid sequences of these *OMP* homologs (Fig. 2). Amino acids sequences are conserved among teleost OMP1 and OMP2 and tetrapod OMP. In particular, the Eph2B-receptor-like loop domain, a potentially key region for OMP function as a molecular switch [35], is highly conserved. Thus, the fundamental structure and physiological function of OMP2 are expected to be similar to those of OMP1 or tetrapod OMP.

**Fig. 3:**
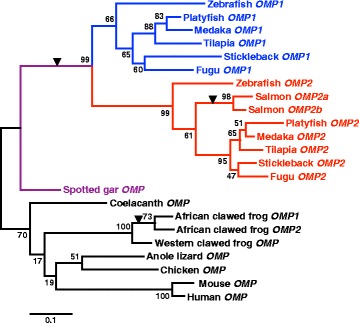
Phylogenetic analysis of *OMP*. Maximum likelihood phylogeny of *OMP*. Colors indicate *OMP* subgroups: blue, teleost *OMP1*; red, teleost *OMP2*; purple, gar *OMP*; black, tetrapod *OMP*. Numbers are bootstrap values for each divergence. Wedges indicate suggested *OMP* duplication events

Based on the genome search, we found that only one OMP exists in gar (Lepisosteus oculatus), which diverged from the teleosts before the occurrence of 3R [12]. These data suggest that the two *OMP*s in teleosts were derived from 3R. To investigate this possibility, we performed phylogenetic analysis that included gar and tetrapods. We constructed a maximum likelihood phylogenetic tree using amino acids sequences from exon 2 of *OMP2* and the homologous regions of *OMP1* and tetrapod *OMP* (Fig. 3). Teleost *OMP* homologs formed a monophyletic group with a bootstrap value of 99 % that consisted of the *OMP1* clade (a bootstrap value of 66 %) and *OMP2* clade (a bootstrap value of 99 %). These data strongly suggest that *OMP* was duplicated in a common ancestor of teleosts, after the divergence of gar. Thus, we suggest that teleost-specific *OMP* duplication was derived from 3R. It should be noted that each of the salmon *OMP*s and xenopus *OMP*s formed a monophyletic group, suggesting that duplication of salmon *OMP*s and xenopus *OMP*s was caused by lineage-specific WGDs that occurred independently in those two lineages.

**Fig. 4:**
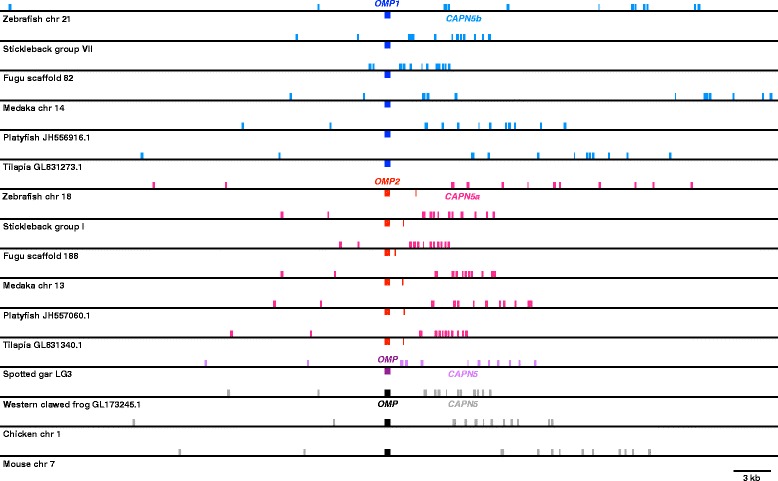
Synteny analysis of *OMP* loci. Exon map was drawn based on Ensembl annotations. Boxes indicate exons: those above the line indicate forward strand-coded; those under the lines indicate reverse strand-coded. Colors indicate *OMP* or *CAPN5* subgroups: blue, teleost *OMP1*; cyan, teleost *CAPN5a*; red, teleost *OMP2*; pink, teleost *CAPN5b*; purple, gar *OMP*; light purple, gar *CAPN5*; black, tetrapod *OMP*; gray, tetrapod *CAPN5*

We next analyzed the synteny of *OMP* loci (Fig. 4) and found that *OMP* is located within the intron of another gene, *Calpain5* (*CAPN5*). Interestingly, teleost *CAPN5*, together with *OMP,* was also duplicated. According to the ZFIN [39], *CAPN5a* are encoded on chromosome 18 and *CAPN5b* are encoded on chromosome 21. Consequently, OMP1 is linked to CAPN5b and OMP2 is linked to CAPN5a. Given that both *OMP1* and *2* are located within intron2 of *CAPN5b* and *a*, respectively, it is highly unlikely that *OMP* duplication was caused by retrotransposition. The genomic structures around *OMP*s (~40 kb) are well conserved between paralogs and also among species except for the coding direction of *OMP*. The results of the synteny analysis support our expectation that the two *OMP*s are derived from 3R.

### OMP2 expression in the retina

**Fig. 5:**
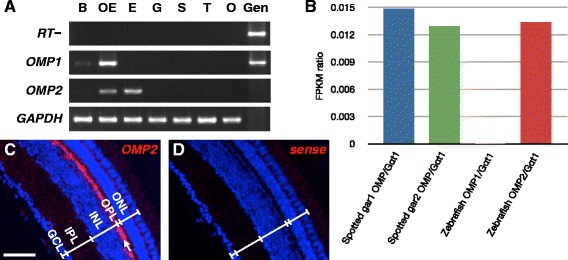
Expression patterns of *OMP1* and *2* at the tissue level. **a** RT-PCR analysis of *OMP1*, *OMP2*, and *GAPDH* (positive control) in adult zebrafish. DNA templates were as follows: B, brain; OE, olfactory epithelium; E, eye; G, gill; S, skin; T, testis; O, ovary; Gen, genomic DNA. RT–, the cDNA synthesis was performed without reverse transcriptase as a negative control for RT-PCR of *OMP1*. Note: When genomic DNA was used as a tmplates, PCR using *OMP2* or *GAPDH* primers did not amplified fragments because of the presence of introns (over 2 kb) in the corresponding sequences in the genomic DNA. **b** Expression of OMP genes in eyes of gar and zebrafish. Bars indicate FPKM ratio of each *OMP* to *G*α*t1*, which is coupling with rhodopsins. The number of spotted gar indicate technical replicate. **c**, **d** Fluorescence *in situ* hybridization analysis for *OMP2* using DIG-labeled antisense riboprobes (**c**) or sense riboprobes (**d**) in transverse sections of adult zebrafish eyes, which were counterstained with DAPI. GCL, ganglion cell layer; IPL, inner plexiform layer; INL, inner nuclear layer; OPL, outer plexiform layer; ONL, outer nuclear layer. Arrow indicates *OMP2*-expressing zone. Scale bar, 50 μm

Three typical fates of duplicated genes are known: pseudogenization, neofunctionalization, and subfunctionalization [3, 18, 19]. There is another fate of duplicated genes, in which gene duplication simply increases the amount of products as represented by the ribosomal DNA genes [40]. However, this is an extreme case in that more than hundred copies exist in the genome. Accodingly, we focus on the possibilities of neofunctionalization and subfunctionalization, which are generally accompanied by differentiated expression patterns that can be assessed by *in situ* hybridization. We thus examined the expression patterns of *OMP1* and *OMP2* (Fig. 5). At first, we investigated the expression of zebrafish *OMP1* and *OMP2* by RT-PCR with total RNA extracted from each organ as template (Fig. 5a). *OMP1* was specifically expressed in the OE, whereas *OMP2* was expressed in the eyes as well as the OE. The expression of *OMP2* in the retina is quite interesting because *OMP* was believed to be specifically expressed in the olfactory organ [24, 27, 36]. To examine whether the expression of *OMP2* in the retina is a common phenomenon among other teleost species, we searched the teleost EST database and found that the expression of *OMP2* is detected in the eyes or the retina of stickleback and tilapia (Table 1), showing that *OMP2* is expressed not only in zebrafish eyes but also in the eyes of some other teleosts. We also examined the expression of *OMP* in spotted gar, of which transcriptome data from the eyes is available. Interestingly, *OMP* was shown to be apparently expressed in the eyes (Fig. 5b). Next, we performed fluorescence *in situ* hybridization with antisense riboprobe (Fig. 5c) or sense riboprobe (Fig. 5d) to *OMP2* to examine expression patterns in detail using cryosectioned eye tissues. Surprisingly, *OMP2*-positive signals were detected specifically in the outermost part (Fig. 5c, *arrow*) of the inner nuclear layer, where retinal horizontal cells are distributed [41].

**Table 1:** Accession number of *OMP2* sequences categorized by tissues from EST database

Species	Olfactory epithelium	Eye or retina	Others or unidentified
Zebrafish	CO801427, CO812065, CO812860, CO958601, DV588230, DV590164, DV594271, DV597000	BF938258, CK352652, CK352729, CK355105, DT863878, DT865346, EB956090	CN317897, CO959420, EH438228, EH442981, EH449085, EH464176, EH468865, EH474918
Stickleback	not found	DW606257	DW626232, DW626233, DW631572
Tilapia	GR669612	GR597376, GR602994	not found

### Divergence of expression patterns between OMP1 and OMP2 in the OE

**Fig. 6:**
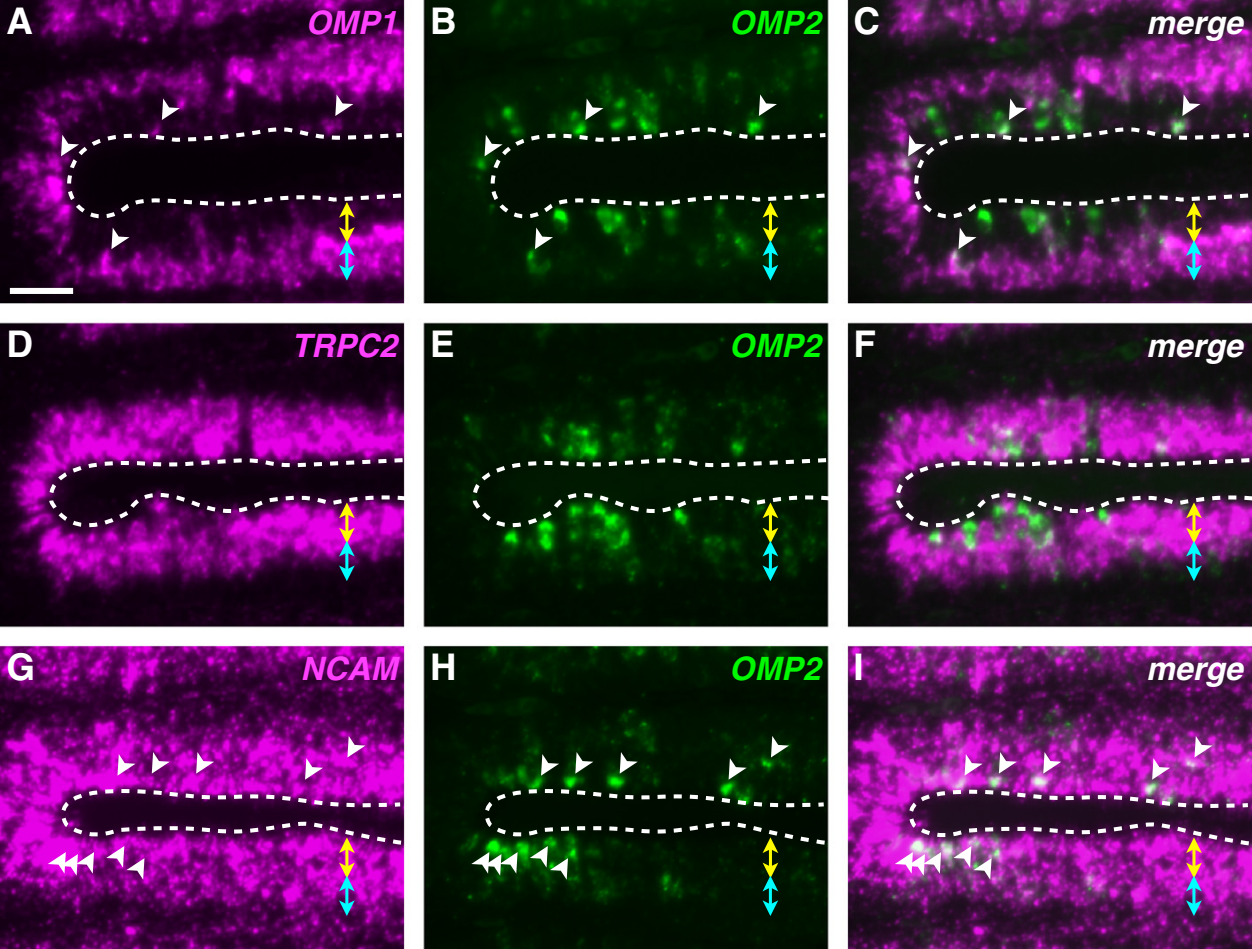
Expression patterns of *OMP1* and *2* at the cellular level in the OE. Two-color fluorescence *in situ* hybridization analysis using DIG- or fluorescein-labeled antisense riboprobes in horizontal sections of the adult zebrafish OE. **a**, **d**, **g** Fluorescent images of Alexa 594 derived from DIG-labeled riboprobes. **b**, **e**, **h** Fluorescent images of Alexa 488 derived from fluorescein-labeled riboprobes. **c**, **f**, **i** Merged images of (**a**) and (**b**), (**d**) and (**e**), and (**g**) and (**h**), respectively. Yellow two-headed arrows and cyan two-headed arrows indicate the superficial layer and the deep layer, respectively. Dashed lines indicate the outlines of the epithelium. White arrowheads indicate cells that coexpress *OMP2* and another gene. Scale bar, 20 μm

RT-PCR analysis (Fig. 5a) showed that both *OMP1* and *2* are expressed in the OE. To investigate these expression patterns at the cellular level, we performed two-color fluorescence *in situ* hybridization using separately labeled antisense riboprobes (Fig. 6a-c). Zebrafish *OMP* (synonym, *OMP1*) was known as a molecular marker for the ciliated OSNs, and was broadly expressed in the deep layer of the olfactory placode (Fig. 6a) as Sato et al. reported previously [28]. In contrast, *OMP2* was sparsely expressed in the superficial layer (Fig. 6b). Merged images show highly exclusive expression of *OMP1* and *2* (Fig. 6c). There were fewer *OMP2*-expressing cells than *OMP1*-expressing cells. These results showed that *OMP1* and *2* are mainly expressed in the deep and superficial layer, respectively, and have distinct expression patterns at the cellular level in the OE. In addition, we noticed that a few cells in the superficial layer expressed both *OMP1* and *2* (Fig. 6a-c, *arrowheads*). Thus, *OMP1* was also expressed infrequently in the superficial layer and the *OMP1*-expressing cells in the superficial layer coexpressed *OMP2*. Previously, Sato et al. [28] also reported that zebrafish *TRPC2* is a marker for the microvillous OSNs, and was expressed in the superficial layer of the OE. We therefore examined the expression of *OMP1* and *TRPC2* (Additional file 1: Figure S1). As expected, *OMP1* was expressed in the deep layer (Additional file 1: Figure S1A), whereas *TRPC2* was in the superficial layer (Additional file 1: Figure S1B). Merged images showed that these genes were not coexpressed (Additional file 1: Figure S1C). We also confirmed that *OMP1* was sparsely expressed in the superficial layer (Additional file 1: Figures S1A-C, arrows). Next, we analyzed the expression of *TRPC2* and *OMP2* (Fig. 6d-f). Merged images showed that expression of these did not overlap (Fig. 6f), indicating that *OMP2* was not coexpressed with *TRPC2*, although both genes were expressed in the superficial layer. The distinctive expression of *OMP2* might suggest that *OMP2*-expressing cells are not OSNs. Thus, we examined the expression of *NCAM*, a neural marker, and *OMP2* (Fig. 6g-i). Merged images showed that *OMP2* was coexpressed with *NCAM* (Fig. 6g-i, *arrowheads*). The results strongly suggest that *OMP2*-expressing cells are actually OSNs.

### G-protein coexpressed with OMP1 and OMP2

**Fig. 7:**
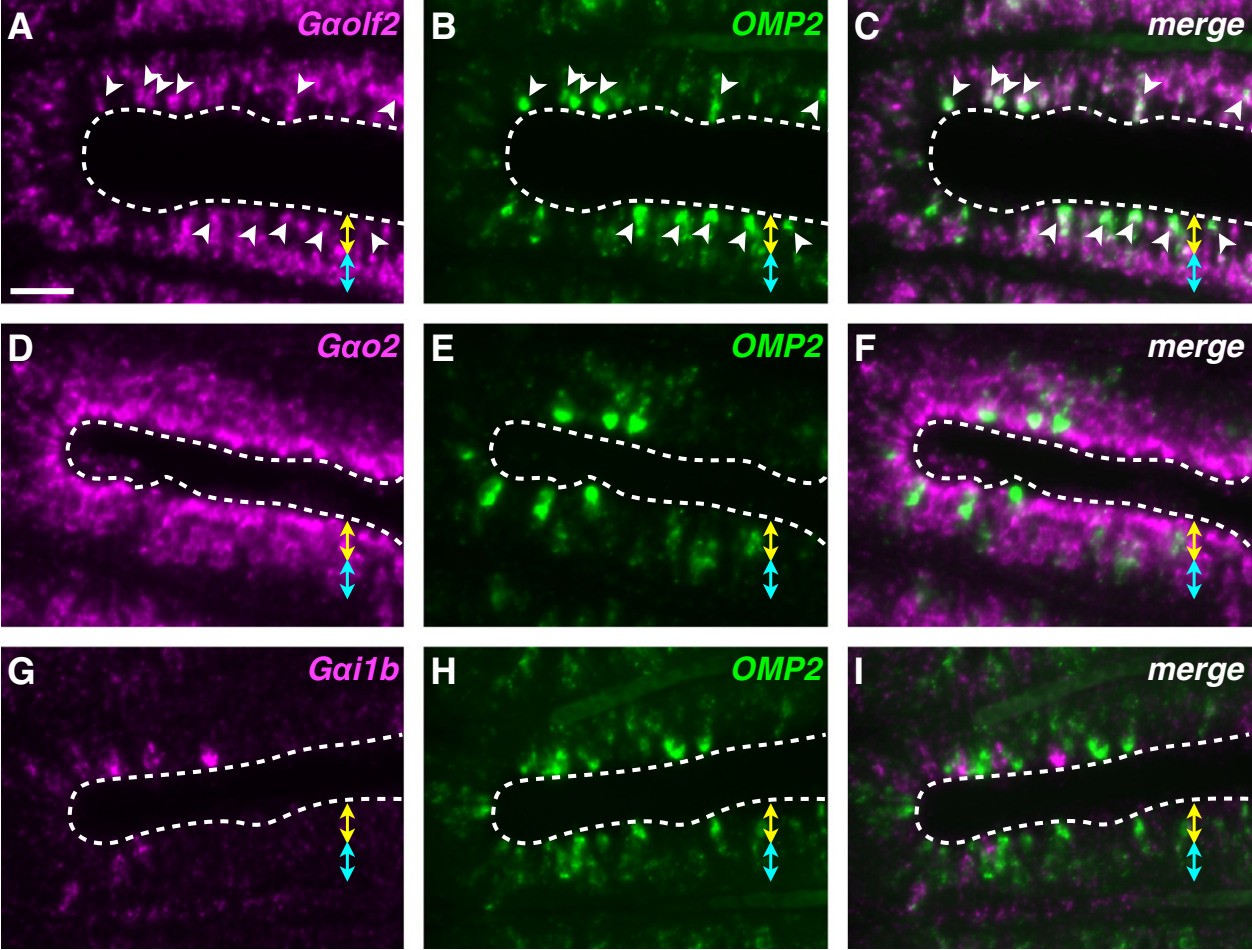
Coexpression of *OMP2* and Gα families. Two-color fluorescence *in situ* hybridization analysis using DIG- or fluorescein-labeled antisense riboprobes in horizontal sections of the adult zebrafish OE. **a**, **d**, **g** Fluorescent images of Alexa 594 derived from DIG-labeled riboprobes. **b**, **e**, **h** Fluorescent images of Alexa 488 derived from fluorescein-labeled riboprobes. **c**, **f**, **i** Merged images of (**a**) and (**b**), (**d**) and (**e**), and (**g**) and (**h**), respectively. Yellow two-headed arrows and cyan two-headed arrows indicate the superficial layer and the deep layer, respectively. Dashed lines indicate the outlines of the epithelium. White arrowheads indicate cells that coexpress *OMP2* and another gene. Scale bar, 20 μm

*OMP2* is expected to be expressed in unidentified OSNs. We are interested in which olfactory receptor genes are coexpressed. Now, four types of olfactory receptor genes are known: odorant receptors (*OR*s) [42], trace amine-associated receptors (*TAAR*s) [43], vomeronasal type 1 receptors (*V1R*s) and vomeronasal type 2 receptors (*V2R*s) [44–46]. However, it is technically hard to examine the coexpression of *OMP2* with receptor genes, because the copy number of them are very large. We thus focused on G-protein α-subunits (*Gα*) genes. It was simply believed that Gαolf is coupled with both ORs [42] and TAARs [43], Gαo is coupled with V2Rs [45, 46], and Gαi2 is coupled with V1Rs [45, 46]. Oka et al. [47] have shown that some *Gα* families are also duplicated in teleosts and are expressed in the OE, namely *Gαolf2*, *Gαo1*, *Gαo2*, and *Gαi1b* (synonym, *gnal, gnao1a, gnao1b,* and *gnaia*, respectively) are expressed in the sensory area of the zebrafish OE. We performed the confirmatory analyses for the expression of the above genes by fluorescent *in situ* hybridization. We also exploratory chose four additional *Gα* genes, *Gαi1a*, *Gαi2a*, *Gαi2b*, and *Gαq* (synonym, *gnai1, gnai2b, gnai2a,* and *gnaq*, respectively), which seem to be well expressed in OE in RT-PCR [47] for the *in situ* hybridization analyses. We were able to detect clear signals for only *Gαolf2*, *Gαo2*, and *Gαi1b*; for the five other genes were not detected (data not shown), probably because the expressions levels of these genes were too low and/or the number of cells expressing these genes were too small. First, we examined the expression of *Gαolf2* and *Gαo2* (Additional file 2: Figure S2). *Gαolf2* was mainly expressed in the deep layer (Additional file 2: Figure S2A), whereas *Gαo2* was expressed in the superficial layer (Additional file 2: Figure S2B). Merged images showed that *Gαolf2* and *Gαo2* were not coexpressed (Additional file 2: Figure S2C). It should be noted that a few *Gαolf2*-expressing cells were in the superficial layer (Additional file 2: Figures S2A-C arrows). The expression patterns of *Gαolf2* and *Gαo2* are similar to those of *OMP1* and *TRPC2* (Additional file 1: Figure S1), respectively. We also confirmed that *OMP1*-positive signals frequently overlapped with *Gαolf2*-positive signals (Additional file 3: Figure S3). Second, we examined the expression of *OMP2* and three *Gα* genes (Fig. 7). Interestingly, *OMP2*-positive signals overlapped with the *Gαolf2*-positive signals in the superficial layer (Fig. 7a-c, *arrowheads*), indicating that *OMP2* was expressed in the *Gαolf2*-expressing cells whose cell bodies were situated in the superficial layer. In contrast, *OMP2* was not expressed in *Gαo2*-expressing cells, although both genes were expressed in the superficial layer (Fig. 7d-f). *OMP2* was not expressed in the *Gαi1b*-expressing cells, which were spottily and sparsely situated in the OE (Fig. 7g-i). These results strongly suggest that *OMP2*-expressing cells coexpress *Gαolf2*. In addition, we examined the coexpression of *OMP2* with *Ora* genes, which are similar to *V1R*s and retained only 6 copies in teleosts [48, 49], but none of them was coexpressed with *OMP2* (Additional file 4: Figure S4).

## Discussion

### Novel insight into the function of OMP in the visual system

**Fig. 8:**
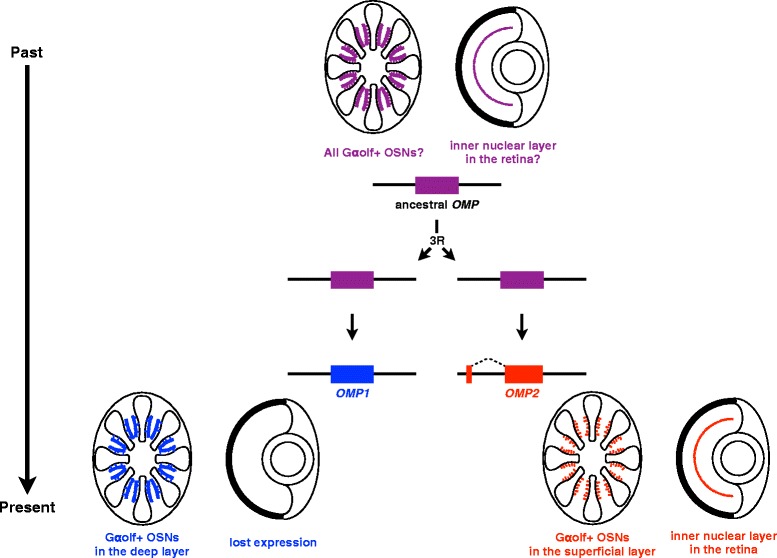
Model of *OMP* evolution. Ancestral *OMP* was expressed in all *Gαolf2*-expressing OSNs and possibly eyes (retina). *OMP1* and *2* emerged after the 3R. Because of subfunctionalization, *OMP1* is now expressed in the deep layer of the OE, whereas *OMP2* is in the superficial layer of the OE and retina

Here we report that the two *OMP*s in teleosts are derived from 3R and have functionally diverged over the ensuing years. We showed the obviously non-canonical expression patterns of *OMP2* in the eyes of broad teleost species (Fig. 5, Table 1). *OMP2* appeared to be expressed in retinal horizontal cells in zebrafish (Fig. 5c). The expression of *OMP2* in the eyes is very interesting because OMP has been believed to be an olfactory organ-specific protein [24, 27, 36]. Unexpected finding of *OMP2* expression in the retina prompted us to investigate the *OMP* of non-teleost fish to understand the ancestral state. We showed that relative expression level of gar *OMP* was as high as that of zebrafish *OMP2* in the eye (Fig. 5b). This data underlies that the *OMP* has been already expressed in the eyes of the common ancestor of bony fish. A recent study reported that *OMP* is expressed in mouse cornea and proposed that *OMP* might be involved in the developmental process of corneal epithelial cells [50]. We searched EST database of mouse and xenopus, only to find no *OMP* sequence from the eye or the retina (data not shown). We also analyzed *OMP2* expression in zebrafish cornea by *in situ* hybridization and detected no *OMP2*-positive signals in the cornea (data not shown). We thus believe that *OMP2* is not expressed in cornea but expressed in retina of zebrafish. Although there is slight discrepancy between mice and zebrafish in that the *OMP* expression is detected in cornea of mice whereas *OMP2* in retina of zebrafish, the expression of *OMP* gene in the visual system is expected to be an ancestral state (Fig. 8, Additional file 5: Figure S5). Based on the above lines of evidence, we propose that the expression of *OMP2* in visual system of teleosts could be explained by subfunctionalization (Fig. 8).

OMP is colocalized with Na^+^/Ca^2+^ exchanger 1 (NCX1) and is involved in the mechanism of Ca^2+^ clearance in mouse OSNs [33]. The *NCX1* ortholog is duplicated in zebrafish, and one of these, *NCX1b*, is expressed in zebrafish eyes as well as in other neural tissues [51, 52]. Thus it is possible that *OMP2* and *NCX1b* are colocalized and are both involved in the regulation of cations in teleost retinal horizontal cells. To further understand the mechanism underlying subfunctionalization in eyes caused by 3R, it is worth examining the gene expressions of *OMP* and *NCX1* of non-teleost fish as well as mice.

### Characterization of OMP1- and OMP2-expressing OSNs

Teleost OE contains three types of OSNs: ciliated, microvillous, and crypt OSNs [53–55]. Generally, the cell bodies of the ciliated OSNs are situated in the deep layer of the OE, whereas those of microvillous OSNs are in the superficial layer. The crypt OSNs reside in the superficial layer of the OE. The axons of these three types of OSNs project to different regions of the olfactory bulb, suggesting that these OSNs have distinct functions [28, 54]. We confirmed that *OMP1* was mainly expressed in the deep layer of the OE (Fig. 6, Additional file 1: Figure S1), and was coexpressed with *Gαolf2* (Additional file 3: Figure S3) but not with *TRPC2* (Additional file 1: Figure S1). These results indicate that *OMP1* corresponds to the previously characterized zebrafish *OMP* [27]. In contrast, *OMP2* was expressed in the superficial layer (Figs. 5 and 6), in which the cell bodies of microvillous and crypt cells are situated. So, we initially considered that *OMP2*-expressing cells might be microvillous or crypt cells. However, this assumption seems unlikely because *OMP2*-expressing cells also express *Gαolf2* (Fig. 7a-c), and neither microvillous nor crypt OSNs express *Gαolf* [54, 55]. Furthermore, we examined the coexpression of *OMP2* with *Ora* genes [48, 49]. In particular, *Ora4* is expressed in zebrafish crypt OSNs [56]. Although these genes were expressed in the OE, none of them was coexpressed with *OMP2* (Additional file 4: Figure S4). Recently, a fourth type of OSN, kappe neuron, was identified, and these neurons are distributed in the superficial layer of the zebrafish OE [57]. They do, however, express *Gαo* [57]. Taken together, these results suggest that the *OMP2*/*Gαolf2*-coexpressing cells are most likely to be ciliated OSNs, in spite of the fact that cell bodies were distributed in the superficial layer. Probably, they also coexpress some *OR*s and/or *TAAR*s. To definitively determine the cell type, a specific antibody against OMP2 is required. Nonetheless, the almost completely non-overlapping expression of *OMP1* and *2* (Fig. 6a-c) implies that *OMP1*- and *2*-expressing cells possess distinct roles in the OE.

### Subfunctionalization between OMP1 and OMP2 in the olfactory system

*OMP2* was expressed in the superficial layer of the OE (Figs. 6 and 7), whereas *OMP1* was mainly expressed in the deep layer (Fig. 6, Additional file 1: Figure S1). The non-overlapping expression of *OMP1* and *2* can be explained by subfunctionalization, which is a model for paralog retention attributed to the reduction and specialization of expression. The 3R derived paralogs of *OMP* in teleosts have partitioned their expression and perhaps function since the WGD event (Fig. 8, Additional file 5: Figure S5). Given that a single *OMP* gene is expressed in all of area of OE in mice and frogs, it is speculated that the single *OMP* gene was expressed in all Gαolf^+^ OSNs in the ancestral group (Additional file 5: Figure S5). In addition, because the OMP is known to play an important and fundamental role in signal transduction in OSNs [e.g. 29], *OMP* could be expressed in all OSNs of OE in the ancestral group that can be assessed by investigating the OE of non-teleost fish. We are now speculating that *OMP* could be expressed in all Gαolf^+^ OSNs in the spotted gar (Additional file 5: Figure S5, shown by blue characters). At present, however, the expression data of spotted gar was lacking, making it difficult to examine in this study.

By focusing on two *OMP*s in teleosts, we proposed the scenarios of subfunctionalization of 3R-derived paralogs. To further verify this scenarios, it is important to incorporate the information about the ancestral states, which are represented by extinct species or close relatives of teleosts. The basal lineages of ray-finned fish (non-teleost fish), which did not undergo the 3R, could be ideal species to infer the ancestral state. Accordingly, the expression pattern of *OMP* in gar and/or polypterus, should be analyzed in detail based on *in situ* hybridization *etc.* in the near future.

### Other duplications of OMP in vertebrate evolution

African clawed frog has two *OMP*s in its genome [36]. Our phylogenetic analysis suggested that xenopus *OMP*s emerged in the African clawed frog lineage. The African clawed frog is an allotetraploid animal [58, 59], and the most recent WGD was estimated to have occurred ~30 MYA [58]. Accordingly, *OMP* duplication in African clawed frog is likely to be derived from a xenopus-specific WGD. Although the two xenopus *OMP*s show distinct expression patterns [36], such expression patterns do not appear to be mutually exclusive. Incomplete differentiation of xenopus *OMP*s is attributed to more recent WGD than 3R. Two *OMP*s are also present in the salmon genome [38]. Our results showed that both salmon *OMP*s are included in the *OMP2* clade. It is well known that another round of WGD occurred independently in the salmon lineage [60, 61]. Therefore, it is most likely that two salmon *OMP2*s found in the present study emerged by this additional WGD. Interestingly, it has been suggested that certain groups of genes tend to be specifically retained after a WGD event [13, 61], and *OMP* would seem to be one of these genes. As salmon genome data become available, it will be interesting to attempt to locate *OMP1* for further analysis.

## Conclusions

We suggested that *OMP* paralogs, which were derived from 3R, have been retained in visual and olfactory system by subfunctionalization (Fig. 8). The expression pattern of *OMP* in gar or polypterus (ray-finned fish without 3R) should be investigated to confirm this scenarios in the future study. In addition, we propose that *OMP2* could be used as a novel molecular marker of OSNs because *OMP1* and *2* were separately expressed in the OE. Thus, the 3R-derived duplicated genes might become promising markers for the classification of various types of cells in the same organ, such as neural tissues.

## Methods

### Ethic statement

The animal protocols and procedures used in this study were approved by the Institutional Animal Care and Use Committee of Tokyo Institute of Technology [62].

### Data mining

Human (*Homo sapiens*), mouse (*Mus musculus*), Western clawed frog, African clawed frog, zebrafish, and salmon *OMP* nucleotides sequences were acquired from DNA Data Bank of Japan (DDBJ) with the ARSA keyword search [63]. Accession numbers are as follows: human, BC069365; mouse, U02557; Western clawed frog, BC061304; African clawed frog, AJ010978, AJ010979; zebrafish, AF457189; salmon, AB490250, AB490251. These sequences were used as queries for a BLASTN search to acquire zebrafish, stickleback, and tilapia *OMP* cDNA sequences from the DDBJ EST database [64]. Accession numbers from the EST database are listed in Table 1. Other *OMP* sequences were acquired from Ensembl genome browser [65] with a TBLASTN search. For all analyses, a BLAST cutoff E-value was set at 1. Then, complete coding sequences were estimated by GeneWise [66]. The same method was used to acquire *CAPN5* sequences. Accession numbers are as follows: human, BC018123; mouse, BC014767; Western clawed frog, BC075496; African clawed frog, BC048218. Information about *OMP* loci for syntenic analysis was also acquired from Ensembl with a BLASTN search.

### Phylogenetic analysis

Deduced amino acid sequences of *OMP*s were aligned by ClustalW2 [67] with default parameters. Because of its low similarity, exon1 of *OMP2* and the homologous regions of the other OMPs were removed from the alignment, and then the maximum likelihood phylogeny was constructed with MEGA6 [68] based on the multiple sequence alignment, using the amino acids WAG + F model with 10,000 bootstrap repetitions and other default parameters.

### RT-PCR

The zebrafish were euthanized under anesthesia using ethyl 4-aminobenzoate. Total RNA was extracted from each organ of two adult zebrafish (strain Tü, 12–24 months old) with TRIzol (Invitrogen). After RNase-free DNase I (TaKaRa) digestion, each RNA sample was diluted to 10 ng/μl. cDNA was synthesized from 100 ng total RNA with SuperScript III Reverse Transcriptase (Invitrogen) using oligo-dT_18_ as a primer for 1 h at 50 °C. Genomic DNA for control was extracted from fins of adult zebrafish with DNeasy Blood & Tissue Kit (QIAGEN). PCR amplification was carried out for 30 s at 94 °C, 30 s at 55 °C, and 40 s at 72 °C for 35 cycles. Sequences of primers are listed in Table 2. To eliminate contamination of the PCR products derived from the genomic DNA, we designed intron-spanning primers for *OMP2* and *GAPDH*.

**Table 2:** PCR primer sequences

Gene	Forward	Reverse
*OMP1*	5’-CAGTCTCTACAACAACGAGGA-3’	5’-TTCATAGGTCTTTAGGAACCC-3’
*OMP2*	5’-ATGGGTTCAGAAATGGAGC-3’	5’-CTAAACAAAGACTACGCATCTGA-3’
*GAPDH*	5’-GGAGTCTTCCTCAGCATTGA-3’	5’-ACAGACTCCTTGATGTTGGC-3’
*TRPC2*	5’-GCGSGAGATYGTGAACA-3’	5’-GACARRTAMGCACGGCTG-3’
*NCAM*	5’-GAGATCAGCGTYGGRGAGTC-3’	5’-ATGTCKGCAGTGGCRTT-3’
*Gαolf*	5’-AAGAAGATMGAGAAACAGTT-3’	5’-TTAAARCACTGAATCCATTT-3’
*Gαo*	5’-ARAGCCATCGAGAARAACC-3’	5’-AGCAYYTGGTCGTATCC-3’
*Gαi*	5’-CAGTCCATMATBGCCATC-3’	5’-GTSTCBGTRAACCACTTGTT-3’
*Gαq*	5’-GGCTCAGGCTATTCAGAAGA-3’	5’-TCTGAAACCAGGGGTATGTT-3’
*Ora1*	5’-GTGTCCCGCAGACTATGACT-3’	5’-ATCCAGATCACGTTATCGATG-3’
*Ora2*	5’-TCCACAATGTGTTTGACGAC-3’	5’-CAGTGAGGTGAAGAAGAGCC-3’
*Ora3*	5’-MAACCTGATGGTGTCGTTG-3’	5’-AAGAGGATGTTGAGMGCCAG-3’
*Ora4*	5’-ACCTGTGTCTGGCTAACCTG-3’	5’-AGCCATGATGACGTGACC-3’
*Ora5*	5’-GTTTTCATCAGACCTCTCGG-3’	5’-TACGGGACAAAACAGGTGTAT-3’
*Ora6*	5’-ATGGTGGATGTGTATGATGTTC-3’	5’-TGATGAAGAACTCCACCTCC-3’

### Transcriptome data analysis

Transcriptome data from the eyes are acquired from DDBJ sequence read archive [69]. Accession numbers are as follows: spotted gar, SRR1288001 and SRR1288144; zebrafish, SRR1562528. Fragments per kilobase of exon per million mapped fragments (FPKM), which reflect relative expression level, were calculated by bowtie-2.2.5 [70] and rsem-1.2.21 [71].

### Riboprobe synthesis

Each zebrafish RT-PCR product was ligated into pBluescript II SK(−) vector. Sequences of primers used for RT-PCR are listed in Table 2. Degenerate primers were designed to amplify several paralogs. After cloning and sequencing, the plasmids were extracted with the QIAfilter Plasmid Midi Kit (QIAGEN) and then linearized with the appropriate restriction enzyme. Digoxigenin (DIG)-labeled or fluorescein-labeled riboprobes were synthesized with T7 or T3 RNA polymerase (Roche) from the linearized plasmids with DIG or fluorescein RNA labeling mix (Roche), respectively. The riboprobes were treated with recombinant DNase I (TaKaRa) to exclude template plasmids.

### Tissue preparation

Olfactory rosettes and eyes of adult zebrafish were dissected out, and fixed in 4 % paraformaldehyde (PFA) in phosphate-buffered saline (PBS) overnight at 4 °C. After fixation, tissues were cryoprotected in 20 % sucrose in PBS, embedded in O.C.T. compound (Sakura Finetek), and sectioned at a thickness of 10 μm on a cryostat (Leica). Sections were stored at −80 °C until use.

### Fluorescence in situ hybridization

Sections were pretreated with 4 % PFA in PBS for 5 min, followed by treatment with 0.3 % H_2_O_2_ in PBS for 15 min and then with 5 μg/ml proteinase K in PBS for 10 min at 37 °C. After fixation with 4 % PFA in PBS for 10 min, sections were treated with 0.2 % glycine in PBS for 5 min, and with 0.2 N HCl for 20 min, followed by 0.25 % acetic anhydride/0.03 N HCl/0.1 M triethanolamine for 3 min. Sections were prehybridized with hybridization solution, which consisted of 50 % formamide; 10 mM Tris–HCl buffer, pH 7.5; 0.6 M NaCl; 1 mM EDTA; 0.25 % SDS; 1× Denhardt’s solution; 5 % dextran sulfate; and 0.2 mg/ml Yeast tRNA, for 40 minutes and were then hybridized with the hybridization solution containing 5 ng/μl DIG-labeled riboprobe at 60 °C overnight. After hybridization, sections were washed sequentially at 50 °C in 5× saline-sodium citrate (SSC), 50 % formamide in 5× SSC (twice), and then in 10 mM Tris–HCl, pH 7.5, containing 150 mM NaCl and 1 mM EDTA (TNE). After RNase treatment with 2 μg/ml RNase A in TNE for 30 min at 37 °C, sections were washed at 50 °C in 2× SSC (twice) and 0.2× SSC (twice). After treatment of the sections with streptavidin/biotin blocking kit (Vector Laboratories) and 1 % blocking reagent (PerkinElmer) in TBS, bound riboprobe was detected with peroxidase-conjugated anti-DIG antibody (1:100; Roche), and visualized with the TSA Plus biotin kit (PerkinElmer) and Alexa 594-conjugated streptavidin (1:500; Molecular Probes). Sections were coverslipped with VECTASHIELD mounting medium with 4’,6-diamidino-2-phenylindole (DAPI) (Vector Laboratories), and images were digitally captured on a fluorescence microscope (Carl Zeiss). In the case of two-color detection, fluorescein-labeled riboprobe was mixed with DIG-labeled riboprobe, and used for hybridization. Fluorescein-labeled riboprobe was detected with peroxidase-conjugated anti-fluorescein antibody (1:500; PerkinElmer), and was visualized with the TSA Plus 2,4-dinitrophenyl (DNP) system (PerkinElmer) and Alexa 488-conjugated anti-DNP antibody (1:500; Molecular Probes). After the detection of the fluorescein-labeled riboprobe, sections were treated with 15 % H_2_O_2_ in PBS for 30 min to inactivate peroxidase. Then, the DIG-labeled riboprobe was detected as described above.

### Availability of data and materials

The data sets supporting the results of this article are available as Additional file.

## Additional files

Additional file 1: Figure S1. Expression patterns of *OMP1* and *TRPC2*. (PDF 4.99 mb) Additional file 2: Figure S2. Expression patterns of *Gαolf2* and *Gαo2*. (PDF 4.99 mb) Additional file 3: Figure S3. Expression patterns of *OMP1* and *Gαolf2*. (PDF 4.99 mb) Additional file 4: Figure S4. Expression patterns of *OMP2* and *Ora* genes. (PDF 4.99 mb) Additional file 5: Figure S5. Schematics of *OMP* duplication and subdivision of gene expressions during evolution. (PDF 4.99 mb)

## Abbreviations

WGD: Whole genome duplication
OE: Olfactory epithelium
OMP: Olfactory marker protein
OSN: Olfactory sensory neuron
KO: Knockout
OR: Odorant receptor
TAAR: Trace amine-associated receptor
V1R: Vomeronasal type 1 receptor
V2R: Vomeronasal type 2 receptor
