# Supplementary material for: Distinct functions of two olfactory marker protein genes derived from teleost-specific whole genome duplication
Source: BMC Evol Biol. 2015 Nov 10;15:245. doi: 10.1186/s12862-015-0530-y (PMC4640105; doi:10.1186/s12862-015-0530-y)
Supplement: Additional file 1: Figure S1. — Expression patterns of OMP1 and TRPC2. (PDF 4.99 mb) [file 12862_2015_530_MOESM1_ESM.pdf]

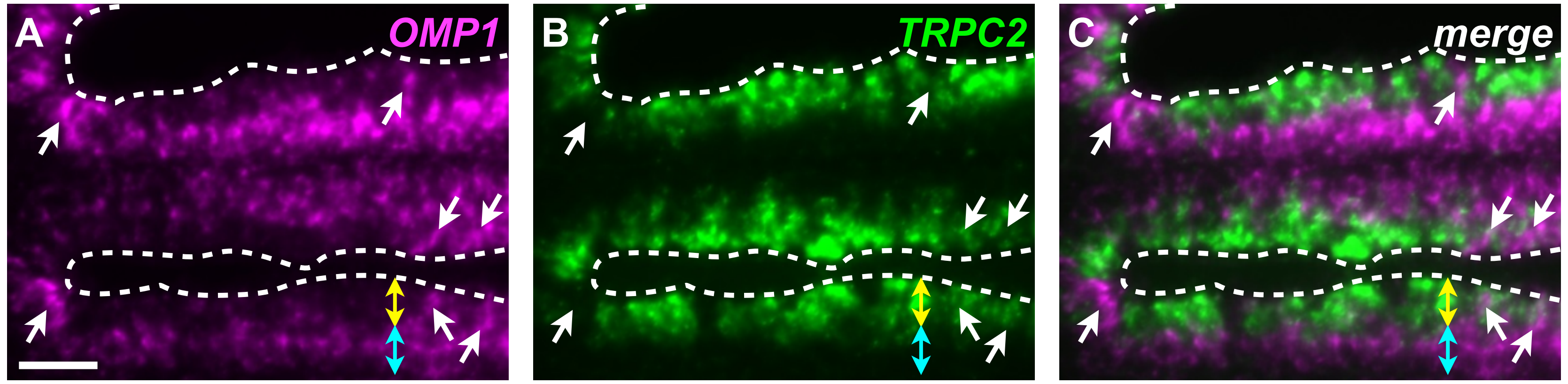

Figure S1 Expression patterns of *OMP1* and *TRPC2*. Two-color fluorescence *in situ* hybridization analysis using DIG- or fluorescein-labeled antisense riboprobes in horizontal sections of adult zebrafish OE. **(A)** Fluorescent images of Alexa 594 derived from DIG-labeled riboprobes. **(B)** Fluorescent images of Alexa 488 derived from fluorescein-labeled riboprobes. **(C)** Merged image of A and B. Yellow two-headed arrows and cyan two-headed arrows indicate the superficial layer and the deep layer, respectively. Dashed lines indicate the outlines of the epithelium. White arrows indicate *OMP1*-expressing cells in the superficial layer. Scale bar, 20 µm.
