# Supplementary material for: Distinct functions of two olfactory marker protein genes derived from teleost-specific whole genome duplication
Source: BMC Evol Biol. 2015 Nov 10;15:245. doi: 10.1186/s12862-015-0530-y (PMC4640105; doi:10.1186/s12862-015-0530-y)
Supplement: Additional file 2: Figure S2. — Expression patterns of Gαolf2 and Gαo2. (PDF 4.99 mb) [file 12862_2015_530_MOESM2_ESM.pdf]

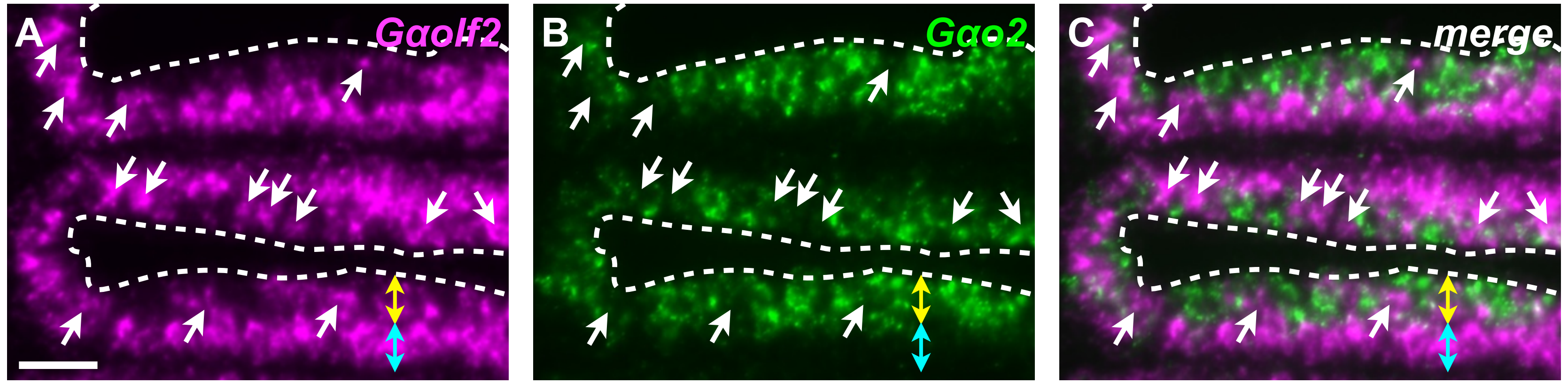

Figure S2 Expression patterns of *Gαolf2* and *Gαo2*. Two-color fluorescence *in situ* hybridization analysis using DIG- or fluorescein-labeled antisense riboprobes in horizontal sections of adult zebrafish OE. **(A)** Fluorescent images of Alexa 594 derived from DIG-labeled riboprobes. **(B)** Fluorescent images of Alexa 488 derived from fluorescein-labeled riboprobes. **(C)** Merged image of A and B. Yellow two-headed arrows and cyan two-headed arrows indicate the superficial layer and the deep layer, respectively. Dashed lines indicate the outlines of the epithelium. White arrows indicate *Gαolf2*-expressing cells in the superficial layer. Scale bar, 20 μm.
