# Supplementary material for: Distinct functions of two olfactory marker protein genes derived from teleost-specific whole genome duplication
Source: BMC Evol Biol. 2015 Nov 10;15:245. doi: 10.1186/s12862-015-0530-y (PMC4640105; doi:10.1186/s12862-015-0530-y)
Supplement: Additional file 4: Figure S4. — Expression patterns of OMP2 and Ora genes. (PDF 4.99 mb) [file 12862_2015_530_MOESM4_ESM.pdf]

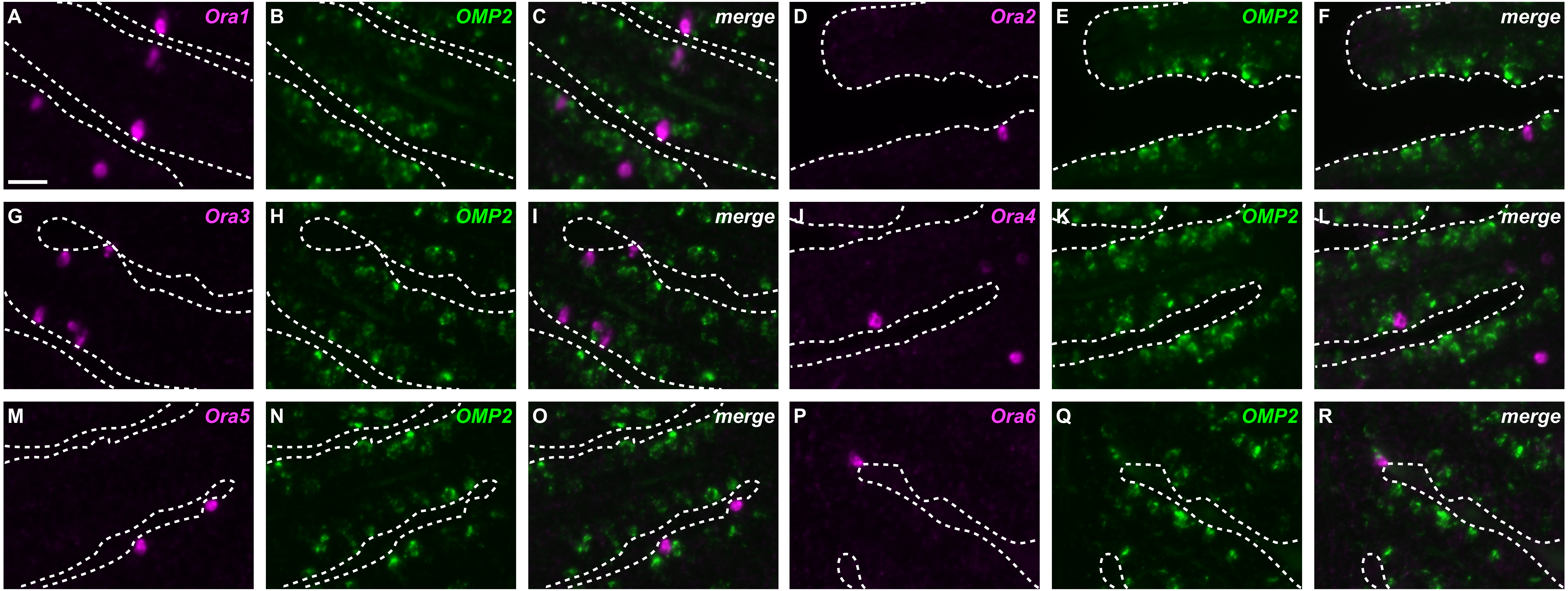

Figure S4 Expression patterns of *OMP2* and *Ora* genes. Two-color fluorescence *in situ* hybridization analysis using DIG- or fluorescein-labeled antisense riboprobes in horizontal sections of adult zebrafish OE. **(A,D,G,J,M,P)** Fluorescent images of Alexa 594 derived from DIG-labeled riboprobes. **(B,E,H,K,N,Q)** Fluorescent images of Alexa 488 derived from fluorescein-labeled riboprobes. **(C,F,I,L,O,R)** Merged images of A and B, D and E, G and H, J and K, M and N, and P and Q, respectively. Dashed lines indicate the outlines of the epithelium. Scale bar, 20 µm.
