# Supplementary material for: Distinct functions of two olfactory marker protein genes derived from teleost-specific whole genome duplication
Source: BMC Evol Biol. 2015 Nov 10;15:245. doi: 10.1186/s12862-015-0530-y (PMC4640105; doi:10.1186/s12862-015-0530-y)
Supplement: Additional file 5: Figure S5. — Schematics of OMP duplication and subdivision of gene expressions during evolution. (PDF 4.99 mb) [file 12862_2015_530_MOESM5_ESM.pdf]

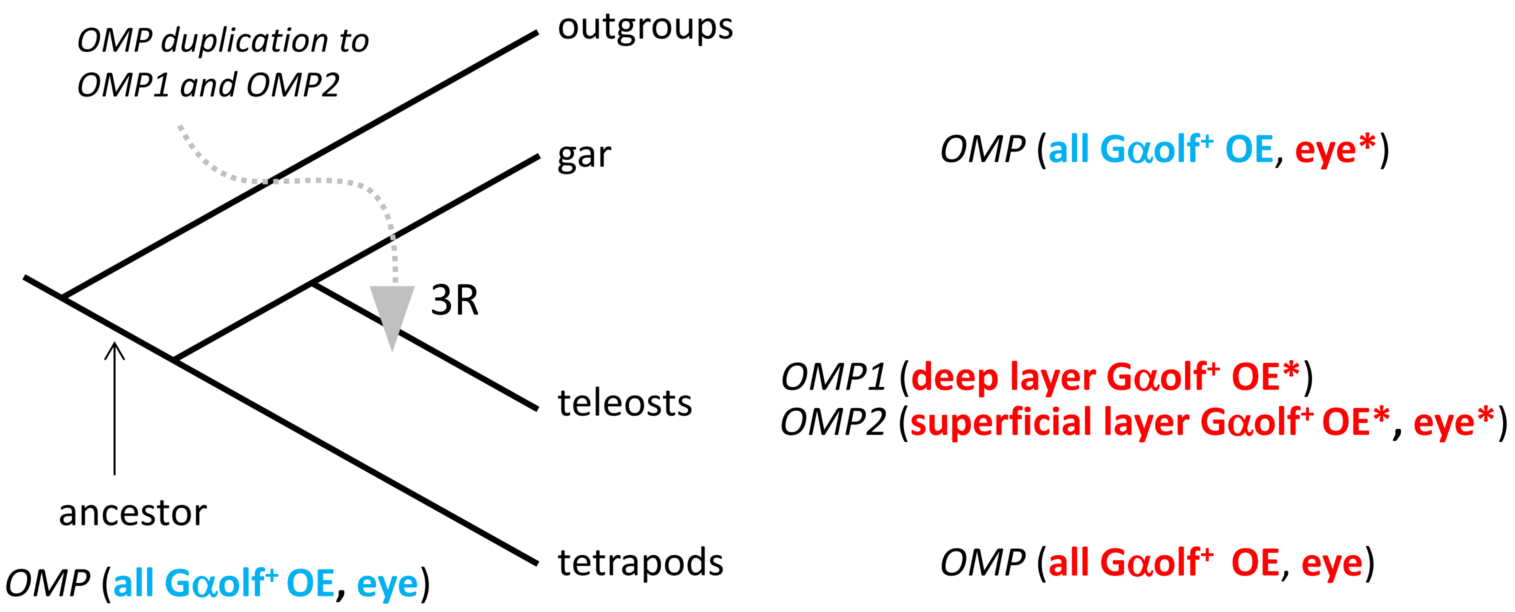

Figure S5 Schematics of *OMP* duplication and subdivision of gene expressions during evolution. The *OMP* was speculated to be expressed in all Gαolf$^+$ OSNs of OE and eyes in ancestral groups. Such ancestral pattern of expression is observed in tetrapods (mouse, frog). The expression of gar OE was speculated as most likely scenario. The expressions of duplicated *OMP*s (*OMP1, 2*) in teleost were subdivided in OE and eyes in that *OMP1* is expressed in deep layer of Gαolf$^+$ OE and *OMP2* in superficial layer of Gαolf$^+$ OE and eyes. The red and blue characters indicate that the expression was examined by experiments and was speculated by the data, respectively. Newly examined expressions in the present study were highlighted by asterisks.
